# Better Quality of Life in Physically Active Adults Regardless of Age

**DOI:** 10.3390/geriatrics9060166

**Published:** 2024-12-19

**Authors:** Magdalena Dąbrowska-Galas, Grzegorz Onik, Magdalena Rutkowska, Iwona Nowakowska

**Affiliations:** 1 Department of Kinesitherapy, Chair of Physiotherapy, Special Methods, School of Health Sciences in Katowice, Medical University of Silesia, 40-752 Katowice, Poland; mrutkowska@sum.edu.pl; 2 Department of Physical Medicine, Chair of Physiotherapy, School of Health Sciences in Katowice, Medical University of Silesia in Katowice, 40-752 Katowice, Poland; 3 Department of Balneoclimatology and Biological Regeneration, Chair of Physiotherapy, School of Health Sciences in Katowice, Medical University of Silesia in Katowice, Medyków 12 Street, 40-752 Katowice, Poland; inowakowska@sum.edu.pl

**Keywords:** adults, physical activity, quality of life, well-being

## Abstract

Quality of life (QOL) has become an important public health issue. Adults presenting better QOL have shown reduced mortality and risk of chronic diseases and better mental health. Regular physical activity (PA) is consistently associated with a number of health benefits in older adults, including betted QOL, which is a key component of healthy aging. The purpose of the study was to examine how physical activity level and age influenced QOL in adults. **Material and Methods**: A total of 378 adults from Poland participated in the study (mean age of 57.89 ± 12.54). Respondents completed questionnaires to measure QOL (WHOQOL-BREF) and physical activity level (International Physical Activity Questionnaire—short form). Linear regression analysis was used to examine the effect of age and PA on QOL. **Results**: Weekly energy expenditure associated with recreational physical activity was correlated with better QOL in all domains: physical (R = 0.5, *p* < 0.001), physiological (R = 0.4, *p* < 0.001), social (R = 0.3, *p* < 0.001) and environmental (R = 0.39, *p* < 0.001). Increases in PA level predicted increases in the physiological domain (β = 0.41, SE = 0.05, *p* < 0.001), social domain (β = 0.34, SE = 0.05, *p* < 0.001) and environmental domain (β = 0.39, SE = 0.05, *p* < 0.001). In the physical domain, increases in both physical activity level (β = 0.41, SE = 0.04, *p* < 0.001) and age (β = 0.31, SE = 0.04, *p* < 0.001) predicted better QOL. **Conclusions**: Our findings emphasize the potential benefits of physical activity on QOL regardless of age. Although aging is combined with various age-related diseases, quality of life improves with age in physically active adults.

## 1. Introduction

According to the World Health Organization (WHO), the population of people aged 60 years and older is expected to increase from 1 billion to 1.4 billion in 2030 and one in six people worldwide will be aged 60 years or older by this time [1]. Aging is associated with various adverse health outcomes; thus, it is very important to take an approach to maintain or improve their quality of life (QOL) [2].

One of the factors influencing quality of life is physical activity (PA), which provides people with many physical and mental benefits [3,4,5,6]. Previous studies have revealed a positive relationship between physical activity and life satisfaction or happiness [3,7,8]. Engaging in regular physical activity reduces the risk of colon cancer, regardless of gender, breast cancer (by 25–30%) and endometrial cancer [8]. It is well known that specific exercises might not only improve quality of life but also reduce risk of falls in older people. Regular practice of amateur dance, such as tango, Mazurek dance and polka, in people of older ages has resulted in an improvement in balance skills, reaction times and motor behavior, cognitive changes and sensory stimulation, which lead to neuro-plasticity [9,10,11]. Regular exercises, including aerobic, balance and proprioceptive components, as well as leg strength training, promote static balance and reduce the risk of falls in the elderly [12]. In turn, lack of physical activity or a sedentary lifestyle are associated with the occurrence of many chronic diseases, such as ischemic heart disease, stroke and diabetes. Inactivity is thought to be a factor that accelerates the decrease in bodily functions and balance control [11,12]. It is considered a modifiable risk factor for the development of malignant tumors [13,14,15]. These diseases contribute to the deterioration of quality of life and a shortening of life expectancy. In Poland, the average life expectancy is about 81.6 years for women and about 73.8 years for men. In 15 countries in Western Europe, life expectancy values are much higher; for example, statistics for men show that life expectancy is almost 8 years shorter in Poland than in Sweden [16].

Quality of life is a cross-disciplinary concept, defined by the World Health Organization (WHO) as the perception of an entity’s position in life in the context of the culture and value systems in which they live and their relationships with their objectives, expectations, standards and interests [17]. It is related to personal satisfaction, happiness and well-being and includes several aspects such as physical and mental health, relationships, leisure and lifestyle [18]. Evidence supports a positive association between physical activity and quality of life at different ages; however, many of these associations are conflicting in different populations and in different age groups [19,20,21]. Most of the authors of the quoted studies considered physical activity as a single factor influencing QOL and stressed the importance of further exploring the association between physical activity and quality of life in different age groups of adults.

In Poland, about 75% of adults feel satisfaction with their lives; however, there is a lack of research on quality of life in Poles. According to previous studies, age, when taken as a single factor, deteriorates QOL, but physical activity improves QOL [22]. Taking into account that previous studies evaluated QOL in different separate age groups, we wanted to fill this gap; thus, the aim of this study was to analyze these two factors together: age and level of physical activity in relation to quality of life in Polish adults.

## 2. Materials and Methods

### 2.1. Participants and Study Design

This study was performed in 2023 in Poland. A total of 400 adults who visited one of three selected healthcare centers were invited to participate in the cross-sectional study using a paper questionnaire or online survey. A single researcher conducted the assessments. Online surveys were provided as an option in case participants wanted to fill out the questionnaire online. Participation in the study was voluntary and anonymous. All recruited adults provided verbal informed consent to participate in the study.

The inclusion criteria were an age > 18, the provision of consent to participate in the research and having no serious illnesses. The exclusion criteria were missing data in a questionnaire.

Data from 378 participants were included in further analyses. The Bioethical Committee of the Medical University of Silesia in Katowice (PCN/022/KB1/147/I/19/20) approved this study.

The mean age of participants was 57.89 years (standard deviation (SD) 12.54 years). There were 135 respondents (35.71%) aged 45–55, 65 respondents (17.2%) aged 56–65 and 178 people (47.09) older than 65. The participants were 69.84% female (246) and 30.16% male (114), were mostly married or in relationships (73.02%, *n* = 276) and had received secondary (51.59%) or higher education (39.42). Most people with a low level of physical activity were from the 45–55 age group (Table 1).

### 2.2. Measurements

The research tool was a survey consisting of three sections: the first included basic socio-demographic data, whereas the two remaining scales were the International Physical Activity Questionnaire—short form (IPAQ-SF) and the WHO quality of life (WHOQOL-BREF). Details of these two internationally used and validated instruments are provided below.

#### 2.2.1. Physical Activity

PA was measured using the International Physical Activity Questionnaire—short form (IPAQ-SF). It is a self-reported questionnaire which consists of questions about the previous 7 days concerning the duration and frequency of low-, moderate- and vigorous-intensity physical activity lasting at least 10 min [23,24].

According to the IPAQ scoring protocol, energy expenditure was expressed as the metabolic equivalent of work (MET)/min (where the metabolic equivalent of 1MET is defined as the amount of oxygen consumed when sitting at rest and is 3.5 mL/O_2_ per kg body weight/min). Total PA MET/week and PA level were calculated in all participants [6,23,24].

#### 2.2.2. Quality of Life

Quality of life was evaluated by a valid and reliable scale: the WHOQOL-BREF. This instrument was developed by the World Health Organization (WHO) and measures 26 items, which are grouped in four domains of quality of life: physical, psychological, social and environmental. All items were rated on a 5-point scale, with each ranging from the highest to lowest score (5–1). Scores of 1 and 5 indicate the lowest negative and highest positive perceptions, respectively [25,26].

### 2.3. Data Analysis

Statistical analysis was performed using the Statistica 13 (Statistica v10 2023, StatSoft, Krakow, Poland). For measurable variables, values such as means, medians, standard deviations and upper and lower quartiles were calculated. Qualitative variables were expressed in percentages. The Shapiro–Wilk test was used to examine the normality of distribution. Comparisons between PA level and QOL domains were made using Kruskal–Wallis analysis of variance. The correlations between total PA MET/week and QOL domains were evaluated with Spearman’s rank correlation coefficient. Linear regression analysis was used to estimate relationships between physical activity level and age on quality of life in different domains. The level of α = 0.05 was assumed as statistically significant.

## 3. Results

### 3.1. Quality of Life 

**Figure 1:**
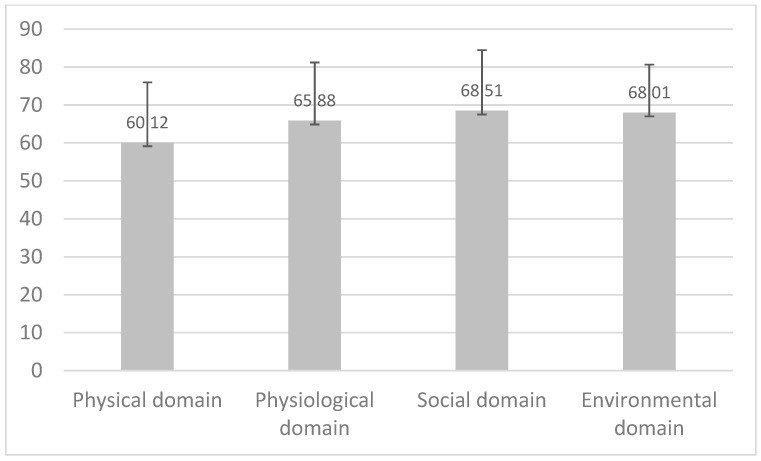
Mean scores and standard deviations of the WHOQOL-BREF in the total sample group (*n* = 378).

Among the 378 participants, the mean age was 57.89 ± 12.54. The mean BMI value was 25.39. According to the WHOWOQ-BREF, the highest score was reported in the social domain (mean 68.51) and the lowest in the physical domain (mean of 60.12) (Figure 1).

### 3.2. Quality of Life According to Physical Activity Level 

**Figure 2:**
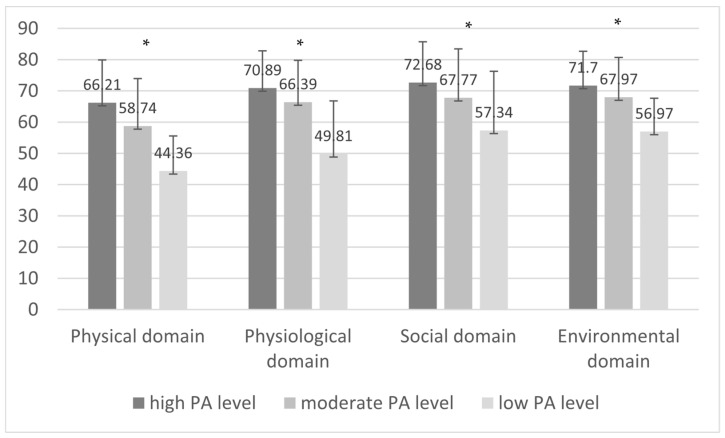
Mean scores and standard deviations of the WHOQOL-BREF and physical activity level using the IPAQ, * *p* < 0.001.

The mean and standard deviation of the physical domain according to PA level were as follows: participants with a high PA level obtained a score of 66.21 ± 13.69 in the WHOQOL-BREF, while adults with a low PA level scored 44.36 ± 11.21 (*p* < 0.001). Statistical differences were reported in all QOL domains and PA levels. The remaining results are shown in Figure 2.

Table 2 shows the correlation between total weekly energy expenditure in physical activity (Total PA MET/week) and QOL domains. The analysis showed a weak positive correlation of total PA MET/week with the physiological domain (r = 0.4, *p* < 0.001), social domain (r = 0.3, *p* < 0.001) and environmental domain (r = 0.39, *p* < 0.001) and a moderate positive correlation with the physical domain (r = 0.5, *p* < 0.001), (Table 2).

According to the regression analysis (Table 3), a higher physical activity level and age predicted better QOL in the physical domain (β = 0.41, *p* < 0.001, and β = 0.31, *p* < 0.001, respectively) and the physiological domain (β = 0.41, *p* < 0.001, and β = 0.22, *p* < 0.001, respectively). In the two remaining domains, age did not predict QOL; however, a higher level of physical activity predicted better QOL in the social domain (β = 0.34, *p* < 0.001) and environmental domain (β = 0.39, *p* < 0.001).

## 4. Discussion

Our paper investigates the role of age and physical activity on quality of life. The results clearly suggest that regular physical activity plays a big role in QOL, regardless of age, that cannot be ignored. In our study, 16.93% of adults reported low or a lack of physical activity.

Jopkiewicz et al. assessed the level of physical activity of Poles aged 20–59. In their study, 79.1% of women and 61.9% of men admitted that their physical activity was irregular and insufficient [27]. Biernat et al. [28] showed that among middle-class Polish workers, at least 32% were physically inactive. In 2015, the percentage of inactive Poles was 35% [29]. Kantanista A et al., when comparing the physical activity levels of Poles, showed that the percentage of physically active adults ranged from 9.2 to 77.6% in men and from 12.0 to 77.6% in women due to the different tools used in the studies [30]. A Eurostat report on physical activity published in March 2018 indicated that nearly half of Europeans do not engage in any physical activity that is beneficial to health; moreover, the number of physically inactive people is increasing [31]. In 2009, 39% of respondents declared a low level of physical activity; in 2013, this rose to 42%, and in 2017, it reached 46%, which in absolute numbers means an increase of almost 35 million physically inactive Europeans. This further means that, currently, around 200 million Europeans do not engage in any health-beneficial physical activity, and 60 million (14%) practice PA rarely and occasionally. About 7% meet WHO recommendations according to the WHO [31].

By increasing the percentage of physically active Poles to a similar level to the five most physically active populations (Austria, Denmark, Germany, Sweden and Finland), the life expectancy of 20-year-old men could be increased by 1.5–1.9 years and 20-year-old women by 1.1–1.5 years in Poland. On the other hand, reducing physical activity to the levels observed in Bulgaria and Romania could reduce life expectancy by 0.6 and 0.4 years among men and women, respectively [31]. We found evidence that higher levels of physical activity are associated with better quality of life in the physical domain (r = 0.5, *p* < 0.001), physiological domain (r = 0.4, *p* < 0.001), social domain (r = 0.3, *p* < 0.001) and environmental domain (r = 0.39, *p* < 0.001). Our findings are in agreement with other results found in the literature. Different tools were used to assess the level of physical activity or quality of life on general adult populations; however, the results showed the importance of physical activity on quality of life, especially physical functioning, vitality, general health and physical and mental components [6,19,27,28,29,30,32,33,34,35,36].

Aging is associated with a deterioration in QOL due to various diseases that are more likely to develop with age [37]; thus, it was necessary to take age into consideration as a factor that, together with PA, may influence QOL. Our results showed that age and PA level were two predictive variables of physical and physiological quality of life. Surprisingly, higher levels of physical activity and older age correlated with better quality of life. The results of many studies confirm the beneficial effect of physical activity on quality of life. At the same time, age contributes to the deterioration of quality of life [37,38,39]. Therefore, it is interesting that the results of our research have shown that physically active people, despite their age, enjoy a better quality of life, especially in physical and physiological domains. On the other hand, a few authors indicated that life satisfaction and happiness increase as people get older because of the presence of more positive emotions and experiences [40,41]. Pucci G. et al. discussed the relationship between physical activity and quality of life in adults. The authors reported a need to further explore the benefit of physical activity in social domains, especially using the WHOQOL [42]. Previous studies showed a lack of or an inconclusive association between PA and QOL in the social domain [19,43,44]. A good-quality social network and relationships are very important factors in the well-being of adults. In our study, the WHOQOL was used, and the results showed that physical activity predicted better quality of life in the social and environmental domains. A weak but positive correlation was noticed in the social domain (r = 0.3, *p* < 0.0001, r = 0.39, *p* < 0.001, respectively). However, age did not show a significant correlation in these two domains.

This study has some limitations that should be considered when interpreting the data. The present sample had a generally healthy profile. There was limited literature that reported on quality of life in Polish adults. This study filled this gap in the literature. Future studies are needed to verify and better understand what other factors influence QOL, especially in social and environmental domains. Some strengths should be mentioned as well. We used the IPAQ to assess PA level and the WHOQOL to evaluate QOL. These are two of the most commonly recommended international tools. This allows us to compare data in the future with other foreign results.

## 5. Conclusions

This paper, in assessing the impact of age and physical activity level on quality of life in adults in Poland, highlights the clear indication that PA is essential for maintaining overall well-being. Our findings emphasize the potential benefits of physical activity on QOL, regardless of age. Although aging is combined with various age-related diseases, quality of life improves with age in physically active adults; thus, every effort should be made to increase the level of physical activity of adults and the percentage of physically active people. Future research is also warranted to explore factors that motivate people to take up physical activity.

## Figures and Tables

**Table 1:** General socio-demographics of the study respondents (*n* = 378).

	*n*	%
Marital status:		
single	73	19.31
married or in a relationship	276	73.02
widowed	29	7.67
Gender:		
female	264	69.84
male	114	30.16
Residence:		
rural	158	41.80
urban	220	58.20
Educational level:		
primary	3	0.79
sector vocational school	31	8.20
secondary	195	51.59
higher	149	39.42
Monthly income per person (PLN):		
<2000	41	10.85
2001–3500	186	49.21
3501–5000	140	37.04
>5001	11	2.91
Physical activity level:		
low	64	16.93
moderate	121	32.01
high	193	51.06
Physical activity level (age 45–55): low moderate high Physical activity level (age 56–65): low moderate high Physical activity level (age > 65): low moderate high	48 36 51 4 18 43 12 67 99	35.55 26.67 37.78 6.15 27.7 66.15 6.74 37.64 55.62

Abbreviations: PLN—Polish Zloty.

**Table 2:** Correlations between total physical activity level using the IPAQ and WHOQOL.

	r	t(*n* − 2)	*p*
	Spearman		
Physical domain and total PA MET/week	0.50	11.05	<0.001
Physiological domain and total PA MET/week	0.40	8.37	<0.001
Social domain and total PA MET/week	0.30	6.18	<0.001
Environmental domain and total PA MET/week	0.39	8.26	<0.001

Abbreviations: PA—physical activity; WHOQOL—World Health Organization quality of life questionnaire; IPAQ—International Physical Activity Questionnaire.

**Table 3:** Predicting quality of life in the physical domain of WHOQOL—regression analysis.

	*B*	SE	β	t	*p*
Physical domain (WHOQOL) F(2.375) = 90,103 *p* < 0.0001, R^2^ adj = 0.321					
Constant term	17.31			4.99	<0.001
Age	0.39	0.04	0.31	7.01	<0.001
Physical activity level	8.71	0.04	0.41	9.44	<0.001
Physiological domain (WHOQOL) F(2.375) = 65,593 *p* < 0.0001, R^2^ adj = 0.255					
Constant term	30.65			8.74	<0.001
Age	0.27	0.05	0.22	4.90	<0.001
Physical activity level	8.27	0.05	0.41	8.87	<0.001
Social domain (WHOQOL) F(2.375) = 24,000 *p* < 0.00001, R^2^ adj = 0.109					
Constant term	53.50			13.37	<0.001
Age	−0.03	0.05	−0.03	−0.55	0.585
Physical activity level	7.27	0.05	0.34	6.84	<0.001
Environmental domain (WHOQOL) F(2.375) = 35,008 *p* < 0.00001, R^2^ adj = 0.153					
Constant term	51.55			16.70	<0.001
Age	0.02	0.05	0.02	0.36	0.716
Physical activity level	6.59	0.05	0.39	8.03	<0.001

Abbreviations: WHOQOL—World Health Organization quality of life questionnaire.

## Data Availability

The raw data supporting the conclusions of this article will be made available by the authors on request.
